# Impact of Sex Steroid Ablation on Viral, Tumour and Vaccine Responses in Aged Mice

**DOI:** 10.1371/journal.pone.0042677

**Published:** 2012-08-03

**Authors:** Tracy S. P. Heng, Jessica J. Reiseger, Anne L. Fletcher, Graham R. Leggatt, Olivia J. White, Katerina Vlahos, Ian H. Frazer, Stephen J. Turner, Richard L. Boyd

**Affiliations:** 1 Monash Immunology and Stem Cell Laboratories, Monash University, Clayton, Victoria, Australia; 2 Department of Cancer Immunology and AIDS, Dana-Farber Cancer Institute, Boston, Massachusetts, United States of America; 3 The University of Queensland Diamantina Institute, Princess Alexandra Hospital, Woolloongabba, Queensland, Australia; 4 Department of Microbiology and Immunology, The University of Melbourne, Parkville, Victoria, Australia; Federal University of São Paulo, Brazil

## Abstract

Recent evidence suggests that the decline in resistance to viral infections with age occurs predominantly as a result of a gradual loss of naïve antigen-specific T cells. As such, restoration of the naïve T cell repertoire to levels seen in young healthy adults may improve defence against infection in the aged. We have previously shown that sex steroid ablation (SSA) rejuvenates the ageing thymus and increases thymic export of naïve T cells, but it remains unclear whether T cell responses are improved. Using mouse models of clinically relevant diseases, we now demonstrate that SSA increases the number of naïve T cells able to respond to antigen, thereby enhancing effector responses in aged mice. Specifically, aged mice exhibit a delay in clearing influenza A virus, which correlates with diminished specific cytotoxic activity. This is due to a decreased magnitude of response and not an intrinsic defect in effector T cell function. Upon SSA, aged mice exhibit increased T cell responsiveness that restores efficient viral clearance. We further demonstrate that SSA decreases the incidence of an inducible tumour in aged mice and can potentially increase their responsiveness to a low-dose human papillomavirus vaccine in clearing pre-formed tumours. As thymectomy abrogates the increase in T cell numbers and responsiveness following SSA, we propose that the T cell effects of SSA are dependent on thymic reactivation and subsequent replenishment of the peripheral T cell pool with newly emigrated naïve T cells. These findings have important implications for strategies to improve protection from infection and responsiveness to vaccination in the aged.

## Introduction

Poor immune competence in the aged is well documented and closely linked to thymic atrophy. The decline in thymic export of naïve T cells is compensated by the expansion of pre-existing memory T cells, restricting the T cell receptor repertoire [1]. Following the development of oligoclonal expansions of CD8^+^ T cells, the ageing peripheral T cell pool exhibits lower diversity for specific antigen recognition and poorer immune responsiveness [2]. These defects manifest as increased susceptibility to infections. For example, influenza A virus infection causes considerable morbidity and mortality in the aged [3] and has been attributed to a reduction in virus-specific CTL activity [4]. Furthermore, success rates following influenza vaccination are often lower in the elderly compared to younger individuals [5]. Thus, strategies that restore immune responsiveness are likely to improve these outcomes following both infection and vaccination.

Sex steroid ablation (SSA) results in the structural and functional regeneration of the aged thymus with increased thymic output and reconstitution of normal numbers of peripheral naïve T cells [6], [7]. Additionally, hormonal modulation of immune function is well established and SSA has been shown to enhance T cell-mediated immunity [8].

In the present study, we used clinically relevant disease models to test the hypothesis that SSA would improve viral, tumour and vaccine responses in aged mice. We found that the diminished capacity of aged mice to respond to influenza A virus infection is not due to intrinsic functional defects but reduced numbers of virus-specific T cells, which can be restored by SSA. Additionally, SSA decreased the incidence of an inducible tumour and we further examined the effects of SSA on the therapeutic efficacy of a human papillomavirus vaccine in clearing pre-formed tumours. Since well-tolerated, reversible SSA regimes currently exist in the clinic, our findings have implications for boosting vaccination success to reduce unnecessary illness and death in the elderly.

## Materials and Methods

### Ethics statement

All animal experimentation and procedures in this study were conducted in accordance with institutional guidelines, and approved by the Alfred Medical Research and Education Precinct Animal Ethics Committee (E/0157/2002/M, E/0298/2004/M, E/0378/2005/M) and the University of Queensland Animal Ethics Committee (CICR/576/04/CICR).

### Animals

Male C57BL/6 6–8 weeks (young), 9 months, 18 months and 24 months (aged) mice, and BALB/c 9 months old mice were obtained from Animal Resources Centre and maintained under specific pathogen-free conditions at Monash University Central Animal Services, Baker Institute Precinct Animal Centre and Princess Alexandra Hospital Biological Research Facility. Mice were acclimatised for 7–14 days prior to experimentation.

### Sex steroid ablation via surgical castration

Mice were anesthetised by inhalation of a gaseous mixture of 3% isofluorane and oxygen, regulated on an anaesthetic machine and maintained via a nose cone. Mice were pre-treated with a subcutaneous injection of the analgesic carprofen (Rimadyl, Pfizer) at a dose of 5 mg/kg of body weight. A small incision was made in the scrotum to expose the testes, which were ligated with absorbable sutures and removed. The wound was closed with absorbable sutures or surgical staples. For surgical stress control, sham castration was performed as above, but without removal of the testes.

### Thymectomy

Mice were anaesthetised by intraperitoneal injection of 5 mg/ml xylazine hydrochloride (Ilium Xylazil-20, Troy Laboratories) and 1 mg/ml ketamine hydrochloride (Parnell) in PBS at a dose of 300 µl/20 g body weight. The upper thoracic cavity was opened to expose the thymic lobes, which were removed by vacuum suction. The wound was closed using surgical staples and anaesthesia was reversed by intraperitoneal injection of atipamezole hydrochloride (Antisedan, Pfizer). At the conclusion of the experiment, the pleural cavity was checked to ensure complete absence of thymic tissue, and mice with thymic remnants were excluded from further analysis.

### MOPC-21 tumour inoculation

Early passage cultures of MOPC-21 cells were recovered from liquid nitrogen and grown to logarithmic phase in RPMI-1640 supplemented with 10% FCS, 100 U/ml penicillin, 100 µg/ml streptomycin, 2 mM GlutaMAX, 50 µM β-mercaptoethanol and 1 mM sodium pyruvate. Each batch was titrated *in vivo* to determine the experimental dose that would result in 80% incidence within 2–3 weeks. 9-month old male BALB/c mice were castrated or sham-castrated 6 weeks prior to MOC-21 inoculation. A small area on the rear flank was shaven and mice were injected subcutaneously with 10^6^ to 5×10^6^ cells in 200 µl of sterile PBS, using a 26 g needle. Mice were monitored 3 times weekly and vernier calipers were used to make perpendicular measurements spanning the largest portion of the tumour in each direction. The mean tumour diameter was calculated as the square root of the product of 2 perpendicular diameters. Experiments were terminated when a tumour grew to a size of 1 cm^2^ in diameter or when 80% of control group develop tumour.

### HPV16 E7GST vaccine immunization

TC-1 cells were maintained as an adherent monolayer in RPMI-1640 supplemented with 10% FCS, 50 U/ml penicillin, 50 µg/ml streptomycin, 2 mM L-glutamine, 1 mM sodium pyruvate and 1 M HEPES buffer. Cells were harvested by trypsinization and washed in PBS several times. 9-month old C57Bl/6 mice were injected subcutaneously with 10^5^ viable cells in 200 µl Dulbecco's PBS in the scruff of the neck, using a 23 g needle, on day 0. Mice were either castrated or sham-castrated on day 5. On day 7, mice were injected subcutaneously in the tail base with either 5 µg or 50 µg of E7GST fusion protein with 10 µg of Quil A saponin adjuvant in 100 µl of Dulbecco's PBS on day 7. Non-vaccinated control mice received Quil A only. Mice were monitored regularly for tumour growth and experiments were terminated when any tumour grew to a size of 1 cm^2^ in diameter (day 25).

### Influenza A virus infection

Mice were anesthetised by inhalation of a gaseous mixture of 3% isofluorane and oxygen and infected intranasally with 10^4^ PFU of A/HKx31 strain of virus in 30 µl. Where indicated castration was performed 6 weeks prior to infection. For memory and secondary challenge experiments, mice were primed by intraperitoneal injection with 1.5×10^7^ PFU of A/PR8 viruses at least 6 weeks prior to challenge with 10^4^ PFU of A/HKx31 strain of virus in 30 µl. The A/HKx31 and A/PR8 influenza viruses differ in their surface hemagglutinin and neuraminidase, but share the PR8 internal proteins (NP, NS1, NS2, M, PA, PB1, and PB2).

### Viral plaque assays

Mice were culled by cervical dislocation and lungs were homogenised and frozen at −70°C until use. To determine viral titres by plaque assay, confluent monolayers of MDCK cells were infected with serial dilutions of lung homogenate for 45 mins at 37°C. 3 ml of L15 media containing 1 mg/ml L-(tosylamido-2-phenyl) ethyl chloromethyl ketone-treated trypsin (Worthington) combined with agarose (0.9%) was added and the cultures were incubated at 37°C, 5% CO_2_ for 3 days. Plaques were then counted by eye and PFU/lung calculated.

### Cell surface labelling

Cells obtained by bronchoalveolar lavage (BAL) were depleted of adherent macrophages by incubation on plastic for 1 h at 37°C. Single-cell suspensions of spleen were enriched for CD8^+^ T cells by incubation on plastic tissue culture plates coated with 200 mg/ml goat anti mouse IgG and IgM antibodies (Jackson ImmunoResearch) for 1 h at 37°C, 5% CO_2_. To detect D^b^NP_366_-specific CD8^+^ T cells, CD8-enriched spleen and macrophage-depleted BAL cells were labelled with PE-conjugated tetrameric complexes of H-2D^b^ and NP_366–374_ peptide (sequence ASNENMETM) at room temperature. Cells were labelled with monoclonal antibodies specific for CD44 (clone IM7), CD4 (clone RM4-5), CD8α (clone 53-6.7), CD62L (clone MEL-14), CD25 (clone PC61) and CD69 (clone H1.2F3) (BD Biosciences) on ice. All samples were washed in ice-cold PBS containing BSA (0.1%) and sodium azide (0.01%) before acquisition on a FACSCalibur using CellQuestPro software (BD Biosciences).

### Peptide stimulation and intracellular cytokine staining

CD8-enriched spleen and macrophage-depleted BAL cells were cultured for 5 h at 37°C in 96-well round-bottom plates at ∼0.5–2×10^6^ cells/well in RPMI-1640 containing 10% FCS, 10 U/ml human rIL-2, and 5 µg/ml GolgiPlug (BD Biosciences), with or without 1 µM NP_366–374_, PA_224–236_ (sequence SSLENFRAYV), or PB1_703–711_ (sequence SSYRRPVGI) peptides (Auspep). The cells were then washed with PBS (containing 0.1% BSA and 0.02% sodium azide) and stained with anti-mouse CD8α-PerCPCy5.5 (clone 53-6.7) for 30 mins on ice. Cells were fixed and permeabilised using the Cytofix/Cytoperm Kit (BD Biosciences), then stained for intracellular cytokine production using anti-mouse IFN-γ-FITC (clone XMG1.2), anti-TNF-α-APC (clone MP6-XT22) and anti mouse IL-2-PE (clone JES6-5H4). Cells were then washed and acquired on a FACSCalibur, using CellQuestPro software. Cytokine-positive cells isolated from unstimulated samples were subtracted from peptide-stimulated samples to determine the number of virus-specific cells.

### Detection of A/HKx31-specific CTL activity

Spleen cells from non-infected C57BL/6 mice were resuspended at 10^8^ cells/ml in HBSS and incubated with 1 µM NP_366–374_ peptide for 1 h at 37°C. Stimulator cells were irradiated at 30Gy, washed twice in HBSS and mixed with equal numbers (3×10^7^) of spleen or BAL cells from Hkx31-infected mice in 40 ml of RPMI-1640 supplemented with 10% FCS, 100 U/ml penicillin, 100 µg/ml streptomycin, 2 mM GlutaMAX, 50 µM β-mercaptoethanol (complete RPMI-10) at 37°C, 5% CO_2_. After 5 days, EL4 target cells were pulsed with 1 µM NP_366–374_ peptide, then labelled with 300 µCi of ^51^Cr (Amersham Biosciences) for 1 h at 37°C. Effector T cells were serially diluted across a range of E∶T ratios in 96-well round-bottom plates. The percentage of specific lysis at 6 h was calculated as 100×(51Cr release from targets with effectors – ^51^Cr release from targets alone)/(^51^Cr release from targets with 1% Triton X-100 – ^51^Cr release from targets alone). The level of ^51^Cr release from targets alone did not exceed 10% of the total ^51^Cr release from targets with 1% Triton X-100.

### Detection of HPV16 E7-specific CTL activity

Spleen cells from young non-immunized C57BL/6 mice were loaded with 0.1 µmol of GF001 peptide, comprising the H-2D^b^-restricted minimal CTL epitope of HPV16 E7 protein (sequence RAHYNIVTF, synthesized in the Frazer laboratory), for 2 h at room temperature. 3.5×10^6^ irradiated stimulator cells were added to 7.5×10^6^ spleen cells from TC-1-inoculated mice per well of a 24-well plate in a total volume of 2 ml of complete RPMI-10 supplemented with 10 U/ml (1 ng/ml) of recombinant mouse IL-2 at 37°C, 5% CO_2_. After 5–7 days, EL4 target cells were resuspended at 5×10^6^/ml, loaded with 1 µmol of GF001 peptide and labelled with 200 µCi of ^51^Cr. Effector cells were serially diluted across a range of E∶T ratios with 3×10^3^ target cells. ^51^Cr release was determined at 4 h and specific lysis was obtained by subtracting unloaded EL4 target cells from sample counts.

### Detection of HPV16 E7-specific IFN-γ secretion by ELISPOT assay

The capture antibody (MabTech) was prepared at 4 µg/ml in freshly prepared filter-sterilized 0.1 M NaHCO_3_ pH 8.4 and 75 µl was added per well of a filter-base 96-well MultiScreen-HA ELISPOT plate (Millipore) at 4°C in the dark overnight. Excess binding sites were blocked by adding 200 µl of complete RPMI-10 for 2 h at room temperature. The plates were washed once with 200 µl of FCS-free RPMI-1640 and 5×10^5^ spleen cells were added per well in 100 µl of complete RPMI-10 supplemented with 100 U/ml of recombinant human IL-2 and varying concentrations of GF001 peptide at 37°C, 5% CO^2^. After 16–20 h, the plates were washed 6 times with 0.02% Tween-20 in PBS (PBST). The biotinylated detection antibody (MabTech) was prepared at 1 µg/ml in PBST containing 2% FCS and 75 µl was added to each well at room temperature in the dark. After 2–4 h, avidin-horseradish peroxidase (Sigma-Aldrich) was diluted to 2.5 µg/ml in PBST/2% FBS and 75 µl was added to each well at room temperature in the dark. After 1 h, the plates were washed 3 times with PBST, then 3 times with PBS. Diaminobenzidine developing reagent (Sigma-Aldrich) was prepared according to manufacturer's recommendations and 75 µl of this substrate solution was added to each well at room temperature. After 1–3 mins or until dark spots appeared, colour development was stopped by thoroughly washing the plates in tap water. The plates were allowed to air-dry overnight. Spots were counted either manually using a camera (Panasonic BP330) mounted on a stereo zoom microscope (Nikon SMZ 10A) with reflected light, or with an automated AID ELISPOT reader with AID software (Autoimmun Diagnostika).

### TCR activation and T cell proliferation assay

Purified NA/LE anti-CD3 (clone 145-2C11) and anti-CD28 (clone 37.51) (BD Biosciences) were diluted to the specified concentrations in sterile PBS. 96-well round-bottom plates were coated with 50 µl of purified anti-CD3 at varying concentrations (1.25 µg/ml, 2.5 µg/ml, 5 µg/ml and 10 µg/ml) and anti-CD28 at a constant concentration (10 µg/ml) in triplicates, sealed with parafilm and incubated at 4°C overnight. To remove excess antibodies, wells were washed twice with sterile PBS. 5×10^5^ spleen cells resuspended in 200 µl of complete RPMI-10 were added to each well in triplicate and incubated in a humidified chamber with 5% CO2 at 37°C for 48 h.

Cells were pulsed with 1 µCi of ^3^H-thymidine (Amersham Biosciences) in a humidified chamber with 5% CO2 at 37°C for 18 h. Cells were harvested onto a glass fibre filter. Liquid scintillant was added to the dried filter and ^3^H-thymidine incorporation was measured on a TopCount micro-plate scintillation counter (Perkin Elmer). Results were expressed as mean CPM for triplicate cultures.

### Statistics

Statistical analysis was performed with the Student's t test (for comparison between 2 groups), One-way ANOVA (for comparison between 3 or more groups) or Fisher exact test (for tumour incidence) using InStat 2 or GraphPad Prism software. A p value of less than or equal to 0.05 was considered significant.

## Results

### Delayed influenza A virus clearance and diminished virus-specific CTL responses in aged mice

To determine the basis of age-associated susceptibility to infection, we utilised a well-characterised mouse model of influenza A virus infection. Intranasal influenza A virus infection of young C57BL/6 causes acute respiratory pneumonia, with cytotoxic T lymphocytes (CTL) playing a major role in limiting and eventually clearing virus around ten days after infection [9], [10]. The primary CD8^+^ T cell response is largely specific for three determinants derived from the nucleoprotein (NP_366–374_ [11]), acidic polymerase (PA_224–233_ [12]) and basic polymerase 1 (PB1_703–711_ [13]).

**Figure 1:**
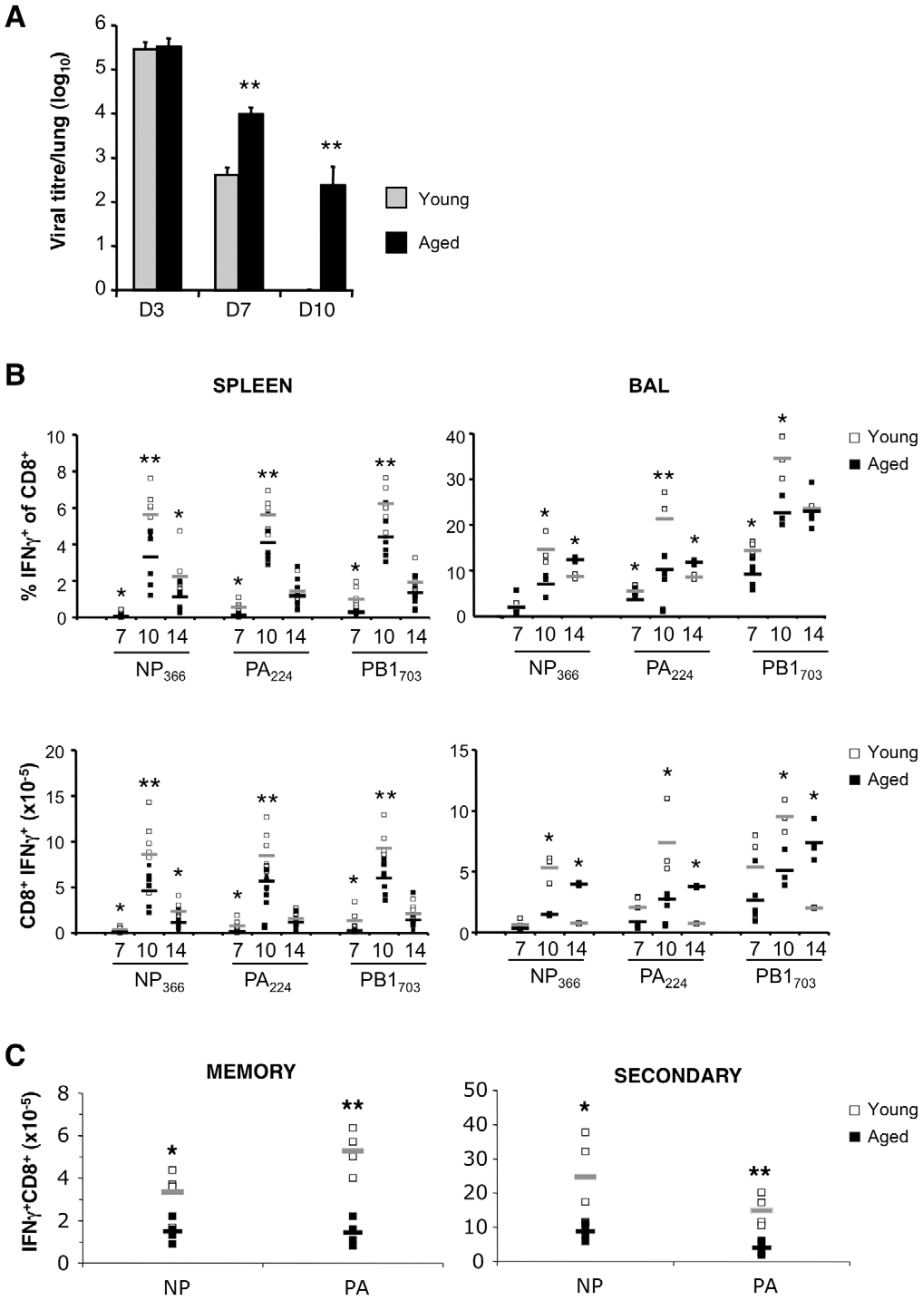
Delayed viral clearance and decreased primary, memory and secondary responses in aged mice. (**A**) Viral titres of 2-mth (Young) and 18-mth (Aged) mice 3, 7 and 10 days after primary intranasal infection with A/HKx31. (**B**) Proportion (top panels) and number (bottom panels) of influenza A viral peptide-specific IFN-γ-expressing CD8^+^ cells in the spleen and BAL 7, 10 and 14 days after infection with A/HKx31. Mean denoted by grey (Young) or black (Aged) bars. (**C**) Number of peptide-specific IFNγ-expressing CD8^+^ cells in the spleen 8 days after priming with A/PR8 (memory response) or challenge with A/HKx31 (secondary response). For secondary challenge, mice were primed with PR8 6 weeks earlier. Results are expressed as mean±SD of 5–6 mice per group per day from 2–3 experiments; *p≤0.05, **p≤0.01 compared to Young (unpaired two-tailed Student's t test).

Intranasal infection with A/HKx31 virus resulted in similar peak viral titres in 2-month (young) and 18-month old mice at day (d) 3 after infection (Fig. 1A). While 18-mth old mice demonstrated significantly higher viral titres at d7 and d10, young mice demonstrated complete viral clearance by d10 after infection. Therefore, as previously reported [14], [15], aged mice demonstrated a diminished capacity to clear influenza A virus infection compared to young mice.

Increased susceptibility to influenza A virus infection has been correlated with a decreased magnitude of CTL responses [14], [15]. We therefore used intracellular cytokine staining to determine whether the delay in viral clearance reflected a decrease in the magnitude and/or dysfunction of responding virus-specific CTLs. In the spleen, 18-mth old mice demonstrated a lower percentage and number of CD8^+^IFN-γ^+^ CTLs compared to young mice for all epitopes tested, especially at d7 and d10 (Fig. 1B, left panels). Similarly in the bronchoalveolar lavage (BAL) fluid, 18-mth old mice had fewer antigen-specific CTLs than young mice at d7 and d10 (Fig. 1B, right panels). Interestingly, at d14 after infection, 18-mth old mice had significantly greater CTL numbers within the BAL when compared to young mice, which likely reflects prolonged antigen availability due to the delay in viral clearance.

The size of the memory CTL pool established after infection has been directly related to the magnitude of the effector CTL response [16], [17]. Given that aged mice demonstrated fewer effector CTL in the spleen during the primary response, aged mice also showed fewer D^b^NP_366_ and D^b^PA_224_-specific CTL in the memory compartment 60 days after infection, which correlated with diminished secondary D^b^NP_336_ and D^b^PA_224_-specific CTL responses (Fig. 1C).

**Figure 2:**
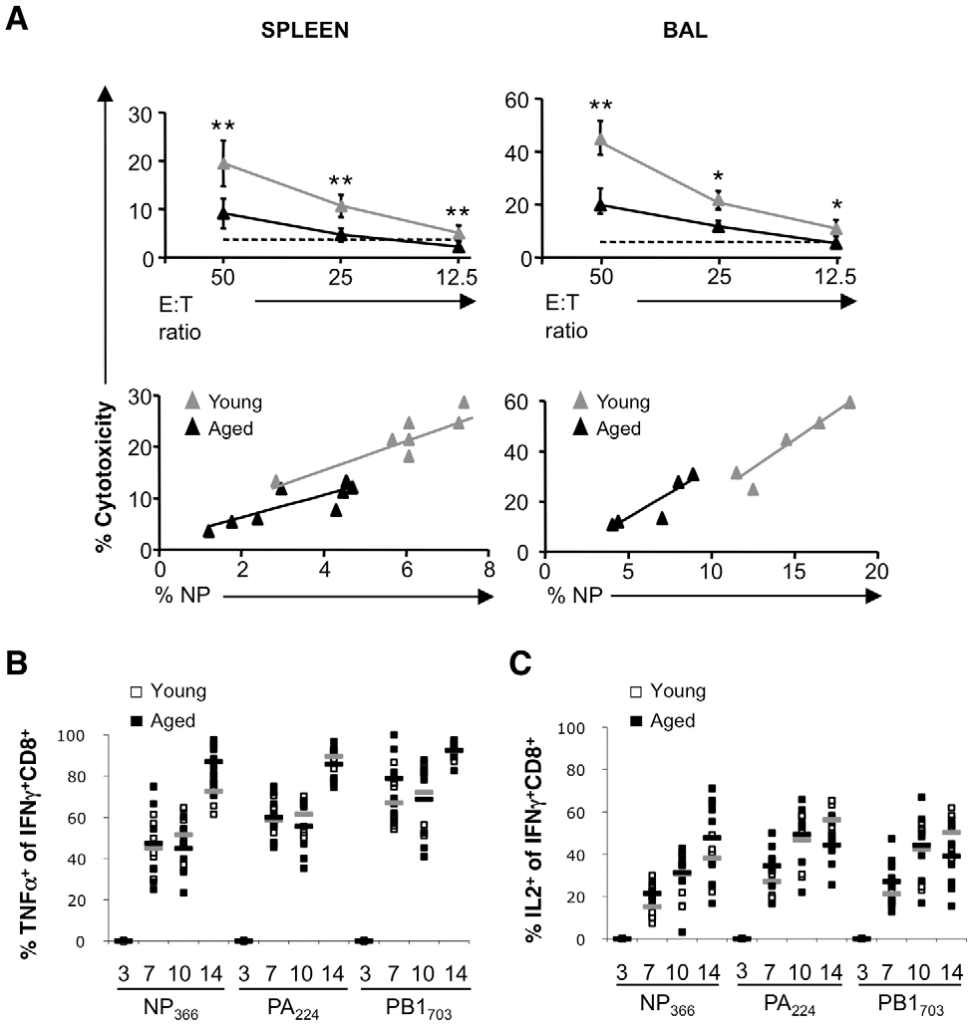
Diminished virus-specific cytotoxic activity in aged mice is associated with decreased CTL numbers rather than reduced functional capacity. (**A**) Cytotoxic activity (top panels) correlated with number of NP_366_-specific CTLs (bottom panels) from spleen and BAL of young and aged mice 10 days after infection. Results are expressed as mean±SD of 5–6 mice per group per day from 2–3 experiments; *p≤0.05, **p≤0.01 compared to Young (unpaired two-tailed Student's t test). (**B**) Percentage of peptide-specific (CD8^+^IFNγ^+^) cells expressing TNFα and (**C**) IL-2 from spleen of young and aged mice 3, 7, 10 and 14 days after infection with A/HKx31. Results were obtained from 6 mice per group per day from 2–3 experiments. Mean denoted by grey (Young) or black (Aged) bars.

CTLs derived from infected aged mice also displayed lower D^b^NP_366_-specific cytotoxicity compared to CTLs derived from young mice at each E∶T ratio measured (Fig. 2A, upper panels). To determine whether decreased cytotoxicity was a result of fewer D^b^NP_366_-specific CTL number or diminished functional capacity per cell, the correlation between cytotoxicity and the proportion of D^b^NP_366_-specific CTLs was determined (Fig. 2A, lower panels). While specific killing strongly correlated with D^b^NP_366_-specific CTL numbers, there was no difference in this correlation between young and aged mice (spleen r = 0.81 vs r = 0.82, respectively; BAL r = 0.88 vs r = 0.88 respectively). Furthermore, we observed no difference in the proportion of influenza A virus-specific CTL producing either TNF-α (Fig. 2B) or IL-2 (Fig. 2C) after *in vitro* peptide stimulation.

Our data indicate that immune deficiency in aged mice is a consequence of reduced epitope-specific CTL numbers and not an intrinsic functional defect. Therefore, if numbers of naïve T cell precursors could be increased prior to vaccination/infection, more robust immunity and improved outcomes to infection would be expected.

### SSA restores virus-specific responses and improves viral clearance

**Figure 3:**
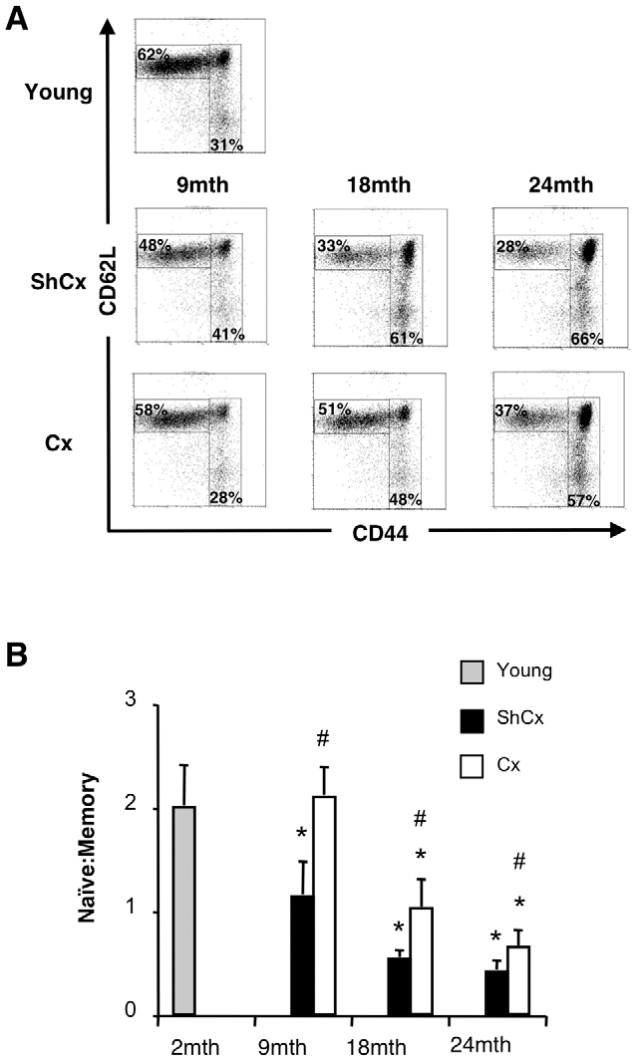
Castration restores the ratio of naïve∶memory T cells in aged mice. (**A**) Representative flow cytometric profiles of naïve and memory CD8^+^ T cells based on CD62L and CD44 expression. Splenocytes were obtained from naïve 2-mth (Young) and 9-, 18- and 24-mth (Aged) mice that were either sham-castrated (ShCx) or castrated (Cx) 6 weeks prior to analysis. (**B**) Results are expressed as mean±SD of 6 mice per group from 2–3 experiments; *p≤0.05 compared to Young; #p≤0.05 compared to ShCx (unpaired two-tailed Student's t test).

In agreement with previous studies [1], [18], we observed a significant age-dependant decline in the ratio of naïve (CD62L^hi^CD44^lo^)∶memory (CD62L^lo^CD44^hi^) CD8^+^ T cells (Fig. 3A). When mice were surgically castrated (Cx) as a means of SSA, an increase in this ratio was observed 6 weeks later in all aged mice compared to their sham-castrated (ShCx) counterparts (Fig. 3B). However, the level of restoration appeared to be limited by age, as the naïve∶memory ratio of 9-mth but not 18-mth or 24-mth old Cx mice was restored to young levels.

**Figure 4:**
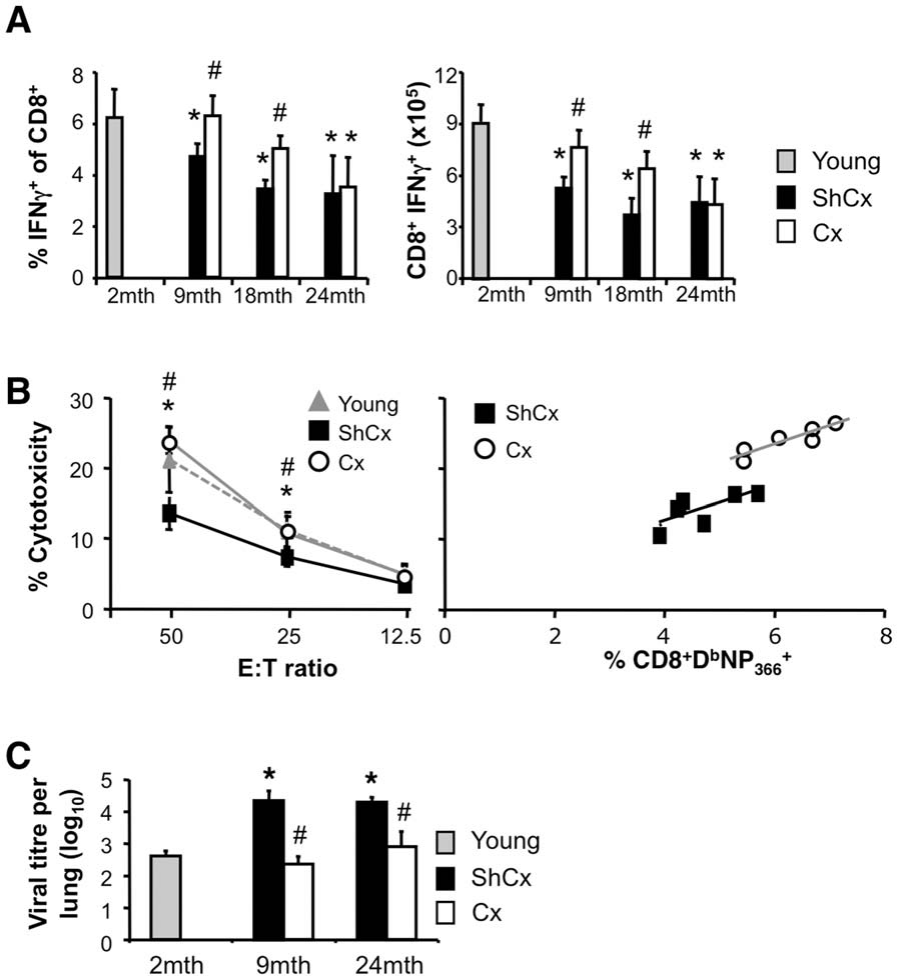
Castration restores virus-specific responses and improves viral clearance. (**A**) Proportion (left) and number (right) of influenza A viral peptide-specific IFN-γ-expressing CD8^+^ cells in the spleen 10 days after infection. (**B**) Cytotoxic activity (left) correlated with number of NP_366_-specific CTLs (right) of 2-mth (Young) and 9-mth (Aged) mice. (**C**) Viral titres 7 days after primary intranasal infection with A/HKx31. Results are expressed as mean±SD of 5–6 mice per group from 2–3 experiments; *p≤0.05 compared to Young; #p≤0.05 compared to ShCx (unpaired two-tailed Student's t test).

To determine whether the restoration of a naïve repertoire would increase resistance to influenza A infection, 9-, 18- and 24-mth old mice were either castrated or sham-castrated 6 weeks prior to infection with A/HKx31 virus. Compared to young mice and in accordance with Fig. 1 and 2, infection of ShCx aged mice elicited fewer D^b^NP_366_-specific CTLs (Fig. 4A). Following castration, 9- and 18-mth old mice showed recovery of both proportion and number of D^b^NP_366_-specific CTLs to young levels (Fig. 4A). Interestingly, 24-mth old Cx mice did not show recovery of D^b^NP_366_-specific CTL numbers, indicating that SSA may have age-limiting effects on improving the response to influenza virus infection.

Analysis of CTL cytotoxicity in 9-mth old mice demonstrated that the castration-induced increase in CD8^+^IFN-γ^+^ T cell numbers corresponded with increased virus-specific cytotoxic activity (Fig. 4B, left). Importantly, the restoration was equivalent to the cytotoxic responses observed in young mice, in contrast to ShCx 9-mth old mice. Again, cytotoxic activity strongly correlated with the number of D^b^NP_366_-specific CTLs (Fig. 4B, right).

To determine whether the subsequent restoration of CTL responses improved viral clearance, lung viral titres were measured at d7 after A/HKx31 infection. ShCx 9- and 24-mth old mice had higher viral titres compared to young mice (Fig. 4C). Importantly, Cx old mice showed improved viral clearance to a level similar to that observed in young mice. In Cx 24-mth old mice, a reduction in lung viral titres occurred despite the observation that their naïve∶memory ratio (Fig. 3B) and virus-specific CTL numbers (Fig. 4A) were not restored to young levels. This suggests that SSA may exert additional effects on non-T cells, such as enhanced antibody response [19] or increased neutrophil production [20], which could contribute to improved viral clearance.

Overall, our data indicate that SSA can increase the number of virus-specific CTLs generated after infection and improve viral clearance in aged mice through beneficial effects that are not limited to T cells.

### Effects of SSA on inducible tumour development and responsiveness of old mice to low-dose vaccination against pre-existing tumour

The enhanced immune responsiveness in Cx aged mice presents significant implications for improving the success of vaccination against viruses and potentially tumours. Early studies have also shown that SSA could delay the development and decrease the incidence of methylcholanthrence-induced tumour [21].

**Figure 5:**
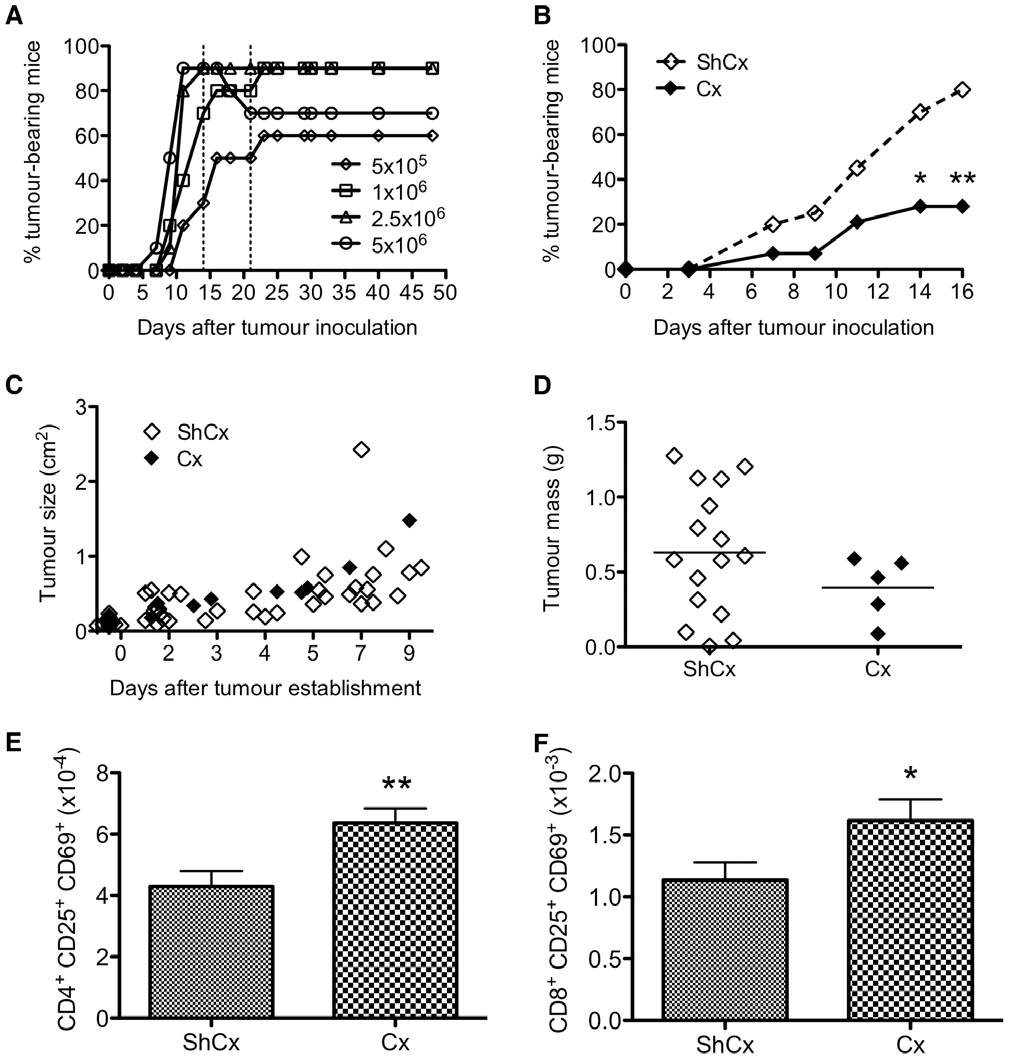
Lower incidence of experimental tumour development in Cx mice. (**A**) Time course of MOPC-21 tumour development. The experimental dose was one that gave 80% incidence in 2–3 weeks, denoted by broken lines. (**B**) Incidence of MOPC-21 tumour development was lower in 9-mth old mice castrated 6 weeks prior to tumour inoculation. Results were obtained from 14–20 mice per group, representative of 2 experiments; *p≤0.05 compared to ShCx (Fisher exact test). (**C**) Growth rate and (**D**) tumour mass in mice that developed tumours. Mean is denoted by black line. (**E**) CD4^+^ and (**F**) CD8^+^ activated T cells in the tumour-draining (inguinal) lymph node. Results are expressed as mean±SE of 8–10 mice per group; *p≤0.05, **p≤0.01 compared to ShCx (unpaired two-tailed Student's t test).

As a first approach to determine the effects of SSA on tumour induction, we utilised the MOPC-21 plasmacytoma cell line [22] to induce solid subcutaneous tumour formation in 9-mth old mice. The dose of MOPC-21 cells was titrated to 80% tumour incidence within 2–3 weeks (Fig. 5A). When 9-mth old mice were castrated 6 weeks prior to tumour inoculation, the incidence of tumour development was dramatically reduced to below 30% (Fig. 5B). However, in mice that develop tumours, we did not observe a delay in the onset of tumour development (Fig. 5B), growth rate (Fig. 5C) or tumour mass (Fig. 5D), indicating that SSA does not counteract growth once the tumour is established. Cx mice also exhibited an increase in activated (CD25^+^CD69^+^) CD4^+^ and CD8^+^ T cells in the inguinal lymph node draining the tumour site (Fig. 5E, F). However, we could not ascertain whether the decrease in tumour incidence in Cx old mice was due to increased activated T cells with anti-tumour effects, as tumour-inoculated mice did not elicit a strong cytolytic response against MOPC-21 targets *in vitro* (data not shown).

Hence, to examine the effects of SSA on anti-tumour T cell function, we utilised a rapidly growing, transplantable mouse tumour cell line (TC-1) expressing the human papillomavirus E7 (HPV16 E7) oncoprotein [23], which is associated with a majority of cervical cancers [24]. The E7GST fusion protein has been shown to retard or reverse the growth of a pre-existing tumour expressing HPV16 E7 by inducing strong E7-specific CTL responses [25].

**Figure 6:**
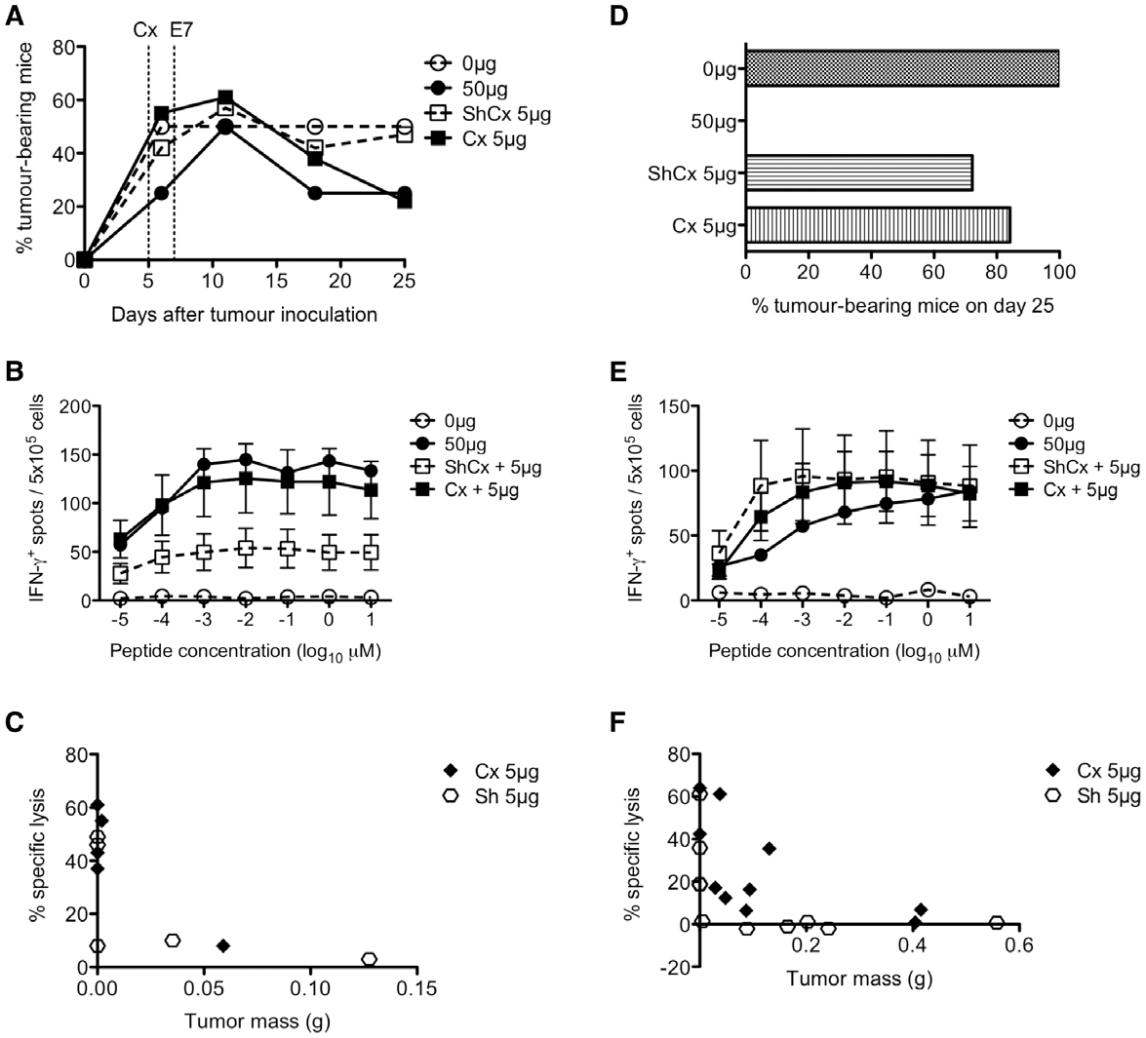
Impact of castration on vaccine-induced tumour clearance. (**A**) Efficacy of E7-induced protection against weakly tumorigenic (50% tumour development) or (**D**) highly tumorigenic TC-1 cells (100% tumour development). In both experiments, 9-mth old mice were either ShCx (open square) or Cx (closed square) 5 days after TC-1 inoculation and received a suboptimal dose (5 µg) of E7 vaccine 2 days after surgery. Control mice either received adjuvant only (Quil A; open circle) or maximum vaccine dose (50 µg; closed circle). On day 25, number of E7-specific (**B, E**) IFN-γ-expressing spleen cells and (**C, F**) cytotoxic activity were determined. Results were obtained from 4–5 mice per control group and 18–19 mice per test group, expressed as mean±SE of 5–10 mice randomly selected per group for ELISPOT. For CTL assay, % specific lysis at 30∶1 effector∶target ratio is shown; mean = 43–52% for 50 µg group, 0% for 0 µg group.

When 9-mth old mice were inoculated with a dose of TC-1 cells that gave rise to 50% tumour incidence, therapeutic vaccination with a sub-optimal dose (5 µg) of E7GST did not cause tumour regression in ShCx mice (Fig. 6A). In Cx mice, however, tumour incidence at day 25 was equivalent to that in mice treated with a 10-fold higher vaccine dose (50 µg). Cx mice also exhibited levels of E7-specific IFN-γ-producing cells (Fig. 6B) and cytotoxic activity (Fig. 6C) comparable to mice that received the maximal vaccine dose. However, when mice were inoculated with a dose of TC-1 cells that gave rise to 100% tumour incidence at day 25, castration did not increase the responsiveness of old mice to the low-dose vaccine (Fig. 6D), nor did it increase the levels of IFN-γ-producing cells (Fig. 6E) and cytotoxic activity (Fig. 6F).

### T cell effects of SSA are dependent on thymic rebound

Our data thus far indicate that SSA can improve immunity in the aged when mice were castrated 4–6 weeks prior to viral challenge (influenza A) or tumour inoculation (MOPC-21). When mice were castrated only 2 days prior to vaccination (HPV16 E7), the effects were more modest or even negligible, depending on tumour load. We therefore propose that the effects of SSA on T cells are more likely directly dependent on thymic regeneration resulting in newly emigrated naïve T cells. This proposition is also based on our previous observation that T cell responsiveness to TCR activation in old mice is increased 6 weeks after castration, concomitant with increased thymic export [7].

**Figure 7:**
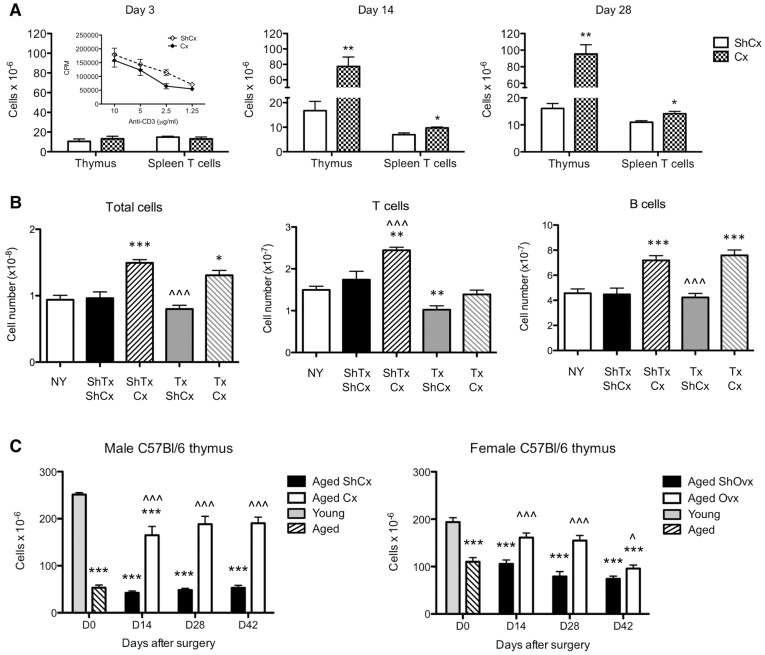
T cell effects of castration in old mice are dependent on thymic regeneration. (**A**) Total thymus cellularity and spleen T cell numbers in 9-mth old mice 3, 14 and 28 days after surgery. Inset show proliferative response of TCR-activated spleen cells from 9-mth old mice 3 days after surgery. Results are expressed as mean±SE of 4 mice per group; *p≤0.05, **p≤0.01 compared to ShCx (unpaired two-tailed Student's t test). (**B**) Total spleen, T cell and B cell numbers in 3-mth old mice 6 weeks after thymectomy. Data is expressed as mean±SE of 6 mice per group; *p≤0.05, **p≤0.01, ***p≤0.001 compared to ShTx ShCx mice; ∧∧∧p≤0.001 compared to Tx Cx mice (One-way ANOVA, Tukey post-test). (**C**) Total thymic cellularity in 2-mth (Young) and 9-mth (Aged) male mice that were either sham-castrated (ShCx) or castrated (Cx), and 9-mth female mice that were either sham-ovariectomised (ShOvx) or ovariectomised (Ovx). Data is expressed as mean±SE of 6–13 mice per group; ***p≤0.001 compared to Young; ∧p≤0.05, ∧∧∧p≤0.001 compared to ShCx or ShOvx (unpaired two-tailed Student's t test).

Accordingly, we did not observe an increase in T cell proliferative response 3 days after castration (Fig. 7A, Day 3 inset). Similarly, increases in thymic cellularity [6] and splenic T cell numbers were observed 2 weeks after castration, but not at 3 days (Fig. 7A). Importantly, when thymectomy (Tx) was performed at the same time as castration, the increase in peripheral T cell numbers 6 weeks after castration was abrogated (Fig. 7B). This decrease was T cell-specific, as TxCx mice still exhibited an increase in B cells. Furthermore, the effects of SSA are not restricted to males, as increased thymic cellularity was also observed by 2 weeks after ovariectomy in aged female mice (Fig. 7C).

In summary, enhanced immune responsiveness in the aged is not an unobtainable goal if clinical interventions are able to improve thymic and T cell function. Our data suggest that SSA may be an effective way of achieving this goal and the timing of therapy is crucial to realise the merits of thymic rejuvenation.

## Discussion

Our results indicate that loss of antigen-specific naïve T cells is a major factor contributing to increased susceptibility to viral infections in the aged. Importantly, we demonstrate that SSA is capable of improving immune protection to infection, at least in part by restoring the naïve T cell pool and increasing antigen-specific T cell precursor frequencies.

Previous studies have attributed decreased D^b^NP_366_-specific CTL response to the loss in number of D^b^NP_366_-specific naïve precursors with age, rather than T cell intrinsic defects [14], [15]. Our data support this concept and extend the observation of diminished responses to a broader spectrum of influenza-derived epitopes in aged mice. The age-related reduction maintained the usual immunodominance hierarchies observed after influenza A virus infection. In this aspect, results from our intracellular cytokine analyses differ from the tetramer studies of Yager *et al* [15], who found an age-related reduction in responsiveness only to NP, but not to PA or PB1 peptides. Nevertheless, our observations support the notion that diminished cellular immunity in the aged is a consequence of general attrition of naïve T cell repertoires.

Previous studies have shown that prolonged influenza A infection in aged mice correlated with impaired CTL activity [26]. We found no difference in the functional capacity (both cytokine and cytotoxicity measured on a per cell basis) between young or aged virus-specific CTLs, which supports the observation that decreased cytotoxic activity is due primarily to limited expansion of virus-specific CTLs rather than intrinsic defects in effector function [14]. Therefore, it is possible that vaccine strategies designed to improve immunity may be limited in the aged due an inability to establish high levels of immunological T cell memory. Vaccination at an early age, on the other hand, establishes robust and durable memory responses [27]. These findings suggest that restoring the naïve T cell repertoire, rather than using novel adjuvants to amplify functional capacity, may achieve more efficacious immune outcomes in the aged.

Attrition of naïve T cell precursors coincides with thymic involution, and the removal of sex steroids has the potent effect of reversing thymic atrophy and restoring thymic output of naïve T cells [7]. Our data indicate that this strategy presents an effective way for restoring naïve T cell repertoires in the aged to enable improved immunity to viral infection.

Interestingly, SSA was not able to restore virus-specific T cell numbers to young levels in extremely old mice (22–24 months). This may be due to limited availability of the appropriate stem cells required for the establishment of the thymic epithelium [28]. While the usefulness of SSA as a way to improve T cell immunity may be limited by the age of patients, it was striking that SSA still resulted in improved viral clearance in these very old mice. It is tempting to speculate that there are benefits in addition to restoration of the naïve T cell repertoires that helps re-establish immune function. For example, we have previously shown that SSA results in enhanced humoral response to hepatitis B vaccine challenge in old mice [19], and increased neutrophil production in patients following haematopoietic stem cell transplantation (HSCT) [20]. Examination of other immune measures of protection, such as NK cell activity, would be of interest in the future.

SSA has also been reported to confer protective effects on tumour induction in very early studies [21]. Similarly, when we utilised a plasmacytoma cell line to induce solid tumours in old mice, SSA decreased the incidence of tumour development and increased activated T cell numbers in the tumour-draining lymph node. It is unlikely that the tumour cell line was hormone-dependent, as MOPC-21 cells were routinely maintained *in vitro* without hormone supplements, and propagated equally well in both female and male mice. SSA appears to only counteract tumour growth at the earliest stages of establishment, as it did not affect the growth rate or mass of individual tumours. It is possible that SSA acts via non-immunological mechanisms, for example by inhibiting angiogenic factors required for the adequate vascularisation of solid tumours.

As MOPC-21 did not elicit a strong cytotoxic T cell response, we turned to the HPV16 E7 oncoprotein-expressing TC-1 cell line to study the impact of SSA on the responsiveness of old mice to therapeutic vaccination. In this model, the E7GST fusion protein has been shown to elicit strong E7-specific CTL responses to cause TC-1 tumour regression in mice [25], therefore allowing us to investigate whether SSA can potentiate E7GST efficacy in retarding or reversing tumour growth. In these experiments, mice were transplanted with TC-1 cells to allow tumour growth, then either sham-castrated or castrated before E7GST treatment. We found that when tumour burden was sufficiently low, the ability of Cx old mice to respond to a sub-optimal dose of E7GST vaccine and produce E7-specific effector T cell function was equivalent to mice that received a vaccine dose that was 10-fold higher.

Roden *et al* [8] has previously shown that castration of young mice increased T cell responsiveness, which persisted when mice were thymectomised 2 weeks prior to castration, suggesting that SSA may exert direct and immediate immunostimulatory effects on the peripheral T cell pool in the absence of a thymus. In old animals however, the true potential of this enhancement may be moderated by the plethora of deficits intrinsic to peripheral T cells [2]. Such limitations might explain the observation that therapeutic vaccination of Cx old mice prior to thymic regeneration did not enhance anti-tumour responses to the point where they could retard tumour growth when tumour load was too high.

The data from the MOPC-21 experiments suggest that castration could inhibit tumour growth and mask any E7GST-induced effects if mice were castrated prior to TC-1 inoculation. Therefore, to examine the impact of SSA on E7GST-induced tumour regression, castration was performed after tumours became palpable and prior to E7GST treatment. Additionally, E7GST was administered within a week of TC-1 inoculation, as TC-1 was a fast-growing tumour. In these experiments when mice were castrated 2 days prior to E7GST peptide vaccination, SSA exerted relatively modest effects compared to when mice were castrated 4–6 weeks prior to A/HKx31 viral infection or MOPC-21 tumour inoculation. Therefore, establishing the contribution of thymic T cell production to the increase in peripheral T cells following SSA is an important point.

In Castro *et al* [21], the protective effects of SSA on tumour induction in young mice were abrogated with thymectomy. In our MOPC-21 studies, our efforts to confirm that the protective effects of SSA were dependent on thymic regeneration have been hampered by low survival rates in old thymectomised mice. However, we have found that thymectomy in younger adult mice abrogated the increase in peripheral T cells that normally occurs following SSA. In addition, we observed an increase in T cell responsiveness to TCR activation in old mice, concomitant with an increase in recent thymic emigrants, 6 weeks after SSA [7], but not earlier. Hence, SSA effects in the T cell compartment are dependent on thymic export and subsequent replenishment of the aged peripheral pool.

The use of surgical castration as a method of SSA has obvious limitations. However, luteinizing hormone-releasing hormone (LHRH) analogues have been used for over two decades in the clinic for to treat prostate cancer, breast cancer and endometriosis. Given the ability of LHRH to block sex steroid production in a reversible manner, the use of this hormone analogue could be applied to rejuvenation of immune capacity in clinical states of immunosuppression [20]. To this effect, we have reported an increase in thymopoiesis and naïve T cell production in prostate cancer and HSCT patients who were treated with LHRH [7], [20]. The present study now indicates that SSA and other approaches that enhance thymic function [18], when strategically timed, could be of great value for boosting immune responsiveness in the aged.
